# The Antifungal and Antibiofilm Activities of Caffeine against *Candida albicans* on Polymethyl Methacrylate Denture Base Material

**DOI:** 10.3390/biomedicines10092078

**Published:** 2022-08-25

**Authors:** Doaa M. AlEraky, Hatem M. Abuohashish, Mohammed M. Gad, Muneer H. Alshuyukh, Amr S. Bugshan, Khalid S. Almulhim, Maha M. Mahmoud

**Affiliations:** 1 Department of Biomedical Dental Sciences, College of Dentistry, Imam Abdulrahman Bin Faisal University, P.O. Box 1982, Dammam 31441, Saudi Arabia; 2 Department of Substitutive Dental Sciences, College of Dentistry, Imam Abdulrahman Bin Faisal University, P.O. Box 1982, Dammam 31441, Saudi Arabia; 3 College of Dentistry, Imam Abdulrahman Bin Faisal University, P.O. Box 1982, Dammam 31441, Saudi Arabia; 4 Department of Restorative Dental Sciences, College of Dentistry, Imam Abdulrahman Bin Faisal University, P.O. Box 1982, Dammam 31441, Saudi Arabia

**Keywords:** caffeine, *Candida albicans*, polymethyl methacrylate acrylic resin (PMMA), denture, stomatitis

## Abstract

Background: In this study, the effect of pure caffeine was established against *Candida albicans* (*C. albicans*) using different microbiological techniques. Methods: Broth microdilution and colony forming units (CFUs) assays were used to detect the minimum inhibitory concentration (MIC) and minimum fungicidal concentration (MFC). The Live/Dead fluorescent dyes were implemented to determine the yeast viability. Polymethyl methacrylate acrylic resin (PMMA) discs were prepared to evaluate caffeine’s effects against adherent *C. albicans* using microplate reader, CFUs, and scanning electron microscope (SEM). Results: caffeine’s MIC was detected around 30 mg/mL, while the MFC was considered at 60 mg/mL. In an agar-well diffusion test, the inhibition zones were wider in caffeine groups. The Live/Dead viability test verified caffeine’s antifungal effects. The optical density of the adherent *C. albicans* on PMMA discs were lower at 620 nm or 410 nm in caffeine groups. CFU count was also reduced by caffeine treatments. SEM revealed the lower adherent *C. albicans* count in caffeine groups. The effect of caffeine was dose-dependent at which the 60 mg/mL dose demonstrated the most prominent effect. Conclusion: The study reinforced caffeine’s antifungal and antibiofilm properties and suggested it as an additive, or even an alternative, disinfectant solution for fungal biofilms on denture surfaces.

## 1. Introduction

*Candida albicans (C. albicans)* is the most frequent fungus that asymptomatically colonizes half of the population worldwide. Yet, the opportunistic mode of this pathogen has been established and associated with several severe infections and a high rate of morbidity and mortality [1]. The virulence factors of *C. albicans* are responsible for the advanced fungal infections in the oral cavity and digestive tract, in addition to mucocutaneous, systemic, and invasive infections [2]. 

Biofilm formation is one of the significant virulence factors of *C. albicans;* the pathogenesis of *C. albicans* is established on the irreversible attachment and construction of the biofilm biomass on the living tissues of the host or on prosthetic devices and implants [3,4]. Adhesion is the initial step of biofilm formation, then morphological changes develop cumulatively by the number of fungus cells and produce extracellular polymeric substances that impact the final biofilm architecture [1,5]. A single species can form biofilm, yet in vivo, combined different species generally underly the formation of biofilm. Previous studies have observed the biofilm maturation in an animal model at 24 h; however, in vitro, it was detected from 38 to 72 h [6,7].

Antifungal agents such as nystatin and miconazole are used to treat mild to moderate candidiasis in the oral cavity for a period of one to two weeks. Oral or intravenous administration of fluconazole is commonly used in case of severe infections [8]. Recently, the prolonged use of fluconazole and its fungistatic, not fungicidal, role has contributed to the growing resistance of *C. albicans* [9]. Studies have shown that the biofilm formation might provoke the candida resistance to antifungals agents [10]. The remarkable ability of *C. albicans* to develop resistance is a public health burden with the limited arsenal of treatment options [11,12] and it is crucial to direct our research studies to explore new antifungal modalities [13]. Notably, the effect of natural compounds on the biofilm activity of drug-resistant *C. albicans* was demonstrated in previous studies [14].

Denture stomatitis is an oral disease that arises due to poor denture hygiene and is generally caused by *C. albicans.* Chlorhexidine (CHX) has been used as a disinfectant agent to diminish the adhesion of *C. albicans* on the denture-based resins [15]. CHX is a biguanide disinfectant [16] that is frequently used in dentistry as an antiseptic oral rinse, mouthwash. CHX has shown a high fungicidal effect and can be used as a topical antifungal agent. However, the proper use of CHX for clinical and dental purposes is important and should be specifically used [15,17].

Caffeine is a natural classical purine alkaloid belonging to the methylxanthines family (1,3,7-trimethixanthine). It is produced in more than eighty herbal species and considered the most common behavior-affecting substance globally [18]. Since the chemical compound has been isolated, pharmaceutical studies have been applied to explore the function of caffeine and the effect of caffeine on the immune and nervous systems has been established [18,19]. Few research studies have reported the antifungal activities of caffeine and recommended further investigations to describe the potential inhibitory effect of caffeine on the growth of *C. albicans* and biofilm formation [20,21]. The aim of our study was to evaluate the antifungal activity of caffeine on *C. albicans* and to assess its antibiofilm activity on polymethyl methacrylate resin (PMMA) denture discs using different microbiological methods. The null hypothesis of the present work is that the antifungal effects of caffeine would not differ from the control.

## 2. Materials and Methods

### 2.1. Chemicals and Materials

Caffeine (Cat. # C080100) was provided from Toronto Research Chemicals Inc. (2 Brisbane Road Toronto, ON, Canada). Live/Dead^®^ yeast viability kit (Cat. # L7009) was purchased from Thermo Fisher Scientific (Waltham, MA, USA). Sabouraud Dextrose Agar (SDA) (Cat. # MM-M-024) and Sabouraud Dextrose Broth (Cat. # MM-M-205) were provided from Molequle-on^®^ (Auckland, New Zealand). Mueller–Hinton Agar (Cat. # CM0337) was provided from Oxoid (Basingstoke, UK), and the 96-well plates were purchased from CELLTREAT (Pepperell, MA, USA). Heat-polymerized acrylic denture base resin (Major.Base.20, Major Prodotti Dentari, Moncalieri, Italy) was used for resin specimens’ preparation.

### 2.2. Antifungal Susceptibility Testing

#### 2.2.1. Broth Microdilution Assay

The antifungal susceptibility testing of caffeine at 15, 30, and 60 mg/mL concentrations was tested against *C. albicans* reference strain (ATCC 10231). *C. albicans* from a glycerol stock was streaked onto SDA plates 2–5 days before the exterminate and the plates were incubated at 37 ± 1 °C for two days. Isolated colonies were inoculated in 20 mL broth media and incubated at 30 ± 1 °C overnight with shaking. The yeast suspension was adjusted at 0.5 McFarland to produce approximately 1 × 10^6^ CFU/mL. The broth microdilution method was performed according to the guidelines from the Clinical and Laboratory Standards Institute (CLSI) M27 protocol for *Candida species* [22] with slight modification [23]. In a 96-well plate, multiple concentrations of caffeine solutions were prepared in sterile phosphate-buffered saline (PBS) by two-fold dilution, 100 µL of each concentration was prepared in triplicate. Then, 100 µL diluted *Candida* (2.5 × 10^3^ CFU/mL) in liquid media was added to each well. CHX (Avalon Pharma, Riyadh, Saudi Arabia) at a concentration of 0.2% was used as a standard antifungal agent also, and positive and negative growth wells were performed as control samples. The microdilution plates were incubated at 37 ± 1 °C, the optical density (OD) of the plate was determined at 620 nm after one and two days using a microplate reader (Bio-Rad xMark TM Microplate Spectrophotometer; Hercules, CA, USA). The minimum inhibitory concentration (MIC) was determined as the lowest concentration at which no turbidity of the yeast growth was observed [24]. To detect the Minimal Fungicidal Concentration (MFC), 10 µL from each well was inoculated on SDA plates and incubated for two days at 37 ± 1 °C and MFC value that was determined at the lowest concentration that showed equal or less than 2 colony-forming units. 

#### 2.2.2. Agar-Well Diffusion

The surface of three SDA plates were inoculated by spreading 100 µL of C. albicans over the entire surface. After this, a 6 mm hole was punched in the agar under aseptic conditions using a microtip. A volume of 50 µL from different concentrations of caffeine, as well as CHX, PBS (control), and distilled water (blank), were added to the wells. Then, the treated plates were incubated overnight at 37 ± 1 °C. After one day, the inhibition zones of caffeine-inoculated wells were visualized and compared with positive- and negative-control wells. The diameter of each inhibition zone was measured (mm) and divided by 2 after subtraction of the diameter of the hole.

#### 2.2.3. Determination of Yeast Viability

The yeast viability was evaluated using the LIVE/DEAD^®^ Yeast Viability Kit (Cat. # L7009) that was purchased from Thermo Fisher Scientific. After one day incubation at 37 ± 1 °C, the yeast suspension with different caffeine concentrations was centrifuged for 5 min at 10,000× *g*. After decanting the supernatant, the remaining pellets were resuspended in another 1 mL of the wash buffer in 1.5 mL tubes. For the cellular staining, 1 µL of FUN^®^ 1 cell stain (Component-A) and 5 µL of Calcofluor^®^ White M2R (Component-B) were added to each yeast suspension sample. Samples were then incubated in the dark for 30 min at 30 °C. Hereafter, 5 µL from each yeast suspension-stained sample was added on a glass slide and covered with a coverslip. The slides were inspected over an inverted fluorescent microscope (Nikon Eclipse Ts2R, Nikon Instruments Inc., Melville, NY, USA). From each sample, four different fields were visualized. Images were taken at excitation/emission wavelength of 488/530 nm and 365/435 nm for the FUN^®^ 1 cell stain and Calcofluor^®^ White M2R, respectively.

### 2.3. Polymethyl Methacrylate (PMMA) Resin Preparation

A total of 60 specimens of polymethyl methacrylate heat-polymerized acrylic resin discs with a thickness of 2 mm, a diameter of 10 mm diameter, and polished surfaces were prepared according to the manufacturer’s guidelines. A digital caliper was used for specimens’ dimensions confirmation, and approved specimens were stored in distilled water for two days at 37 ± 1 °C before testing procedures. The PMMA discs composed five groups (*n* = 10), as follows: Group 1: control group containing PBS; Group 2: the standard (CHX); Group 3: caffeine 60 mg/mL (CAF-60); Group 4: caffeine 30 mg/mL (CAF-30); and Group 5: caffeine 15 mg/mL (CAF-15).

### 2.4. Antibiofilm Activity Assay

Under aseptic conditions in the laminar flow safety cabinet, each disc was sterilized with 95% ethanol on each side then subjected to 1 h ultraviolet illumination. The discs were placed in 12-well plates according the 5 groups with 1 mL of artificial saliva and incubated for 2 h. The overnight culture of *C. albicans* was adjusted to 0.5 McFarland, and 1 mL of yeast suspension was added to each well with different concentration of caffeine, except for the control samples. The plates were incubated at 37 ± 1 °C for two days to allow the biofilm formation phase of *C. albicans*. Nonadherent cells were removed by washing the discs twice with PBS and placed in new, sterile, 12-well plastic plates. Finally, the biofilms were scraped and vortexed for 2 min at 3000 rpm speed to dislodge the adherent cells from the denture [25,26]. The adherent fungal cells were evaluated by microplate reader (Bio-Rad xMark TM Microplate Spectrophotometer, Hercules, CA, USA) at 620 and 410 nm. 

### 2.5. Colony Forming Unit Assay

To assess the antibiofilm activity, Colony Forming Unit Assay was also performed. A volume of 100 μL of each well was inoculated on SDA plates and incubated at 37 ± 1 °C; the counting of colonies was performed within one day to easily distinguish the colonies before the overgrow and after two days to allow scoring of any slow grow isolates. After two days of incubation at 37 ± 1 °C, the colonies were then captured and counted using an Image J 1.37b image analysis system (National Institutes of Health, Bethesda, MD, USA). The experiment was independently performed by two microbiologists using three replicates’ plates to ensure the reproducibility.

### 2.6. Scanning Electron Microscope

One randomly selected disc per group was used for the scanning electron microscope (SEM) procedure. The PMMA discs were initially fixed at room temperature in 2.5% glutaraldehyde solution. The discs were then dehydrated in ascending ethanol concentrations. Afterwards, the specimens were gold sputter-coated after mounting on metallic stubs (Quorum, Q150R ES, Lewes, UK). Samples were inspected under SEM instrument (Emcrafts, Gyeonggi-do, Korea) at 10kV at ×2000 magnification.

### 2.7. Statistical Analysis

Graph Pad Prism V-5 was employed as statistical software (GraphPad Software, Inc., La Jolla, CA, USA). All numerical values were presented as arithmetic means (±) their standard deviations (SD). After testing the normality with the Shapiro–Wilk test, one-way analysis of variance (ANOVA) followed by Turkey’s post hoc test were used for data with normal distribution (microdilution analysis). Data without normal distribution (CFU and Agar-well diffusion) were analyzed using Kruskal–Wallis test and Dunn’s post hoc test. The statistical significance for all analyses was considered when *p* ≤ 0.05.

## 3. Results

**Figure 1:**
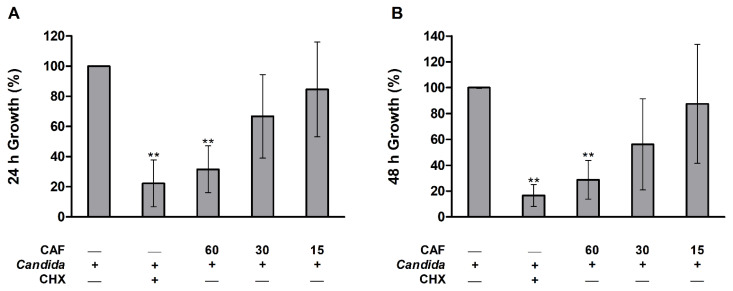
Microdilution test for caffeine (CAF) effects against *C. albicans* growth showing the percentage (%) growth calculated from the measured optical density (OD) of different groups measured at 620 nm. (**A**) After 24 h incubation at 37 °C with broth media. (**B**) After 48 h incubation at 37 °C with broth media. Data are expressed as mean ± SD (*n* = 6) and statistically analyzed using one-way ANOVA followed by Tukey’s post hoc test. The significance was considered when ** *p* ≤ 0.01 as compared with control.

Results of the microdilution test showed that caffeine at the 30 mg/mL concentration showed no visible turbidity of the yeast growth (MIC). Moreover, the results of the optical density using plate reader are presented in Figure 1. The growth of *C. albicans* was significantly (*p* ≤ 0.01) reduced in CHX and caffeine (60 mg/mL) groups as compared to the control group following 24 h of incubation with growth media (Figure 1,A,B). The 30 and 15 mg/mL concentrations of caffeine did not demonstrate a statistically significant decrease in the percentage of *C. albicans* growth compared to the control group (Figure 1A,B). 

**Figure 2:**
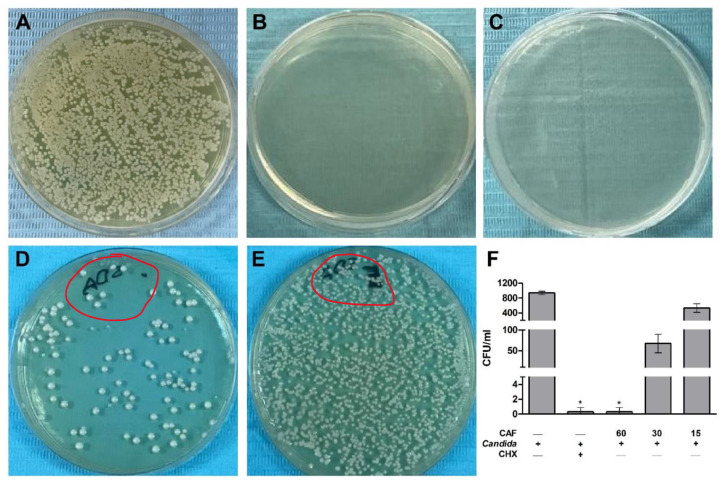
Antifungal Susceptibility Assay of caffeine (CAF) against *C. albicans* growth. (**A**) Fungal cell overgrowth in control group (PBS) containing no treatments. (**B**) Complete inhibition of the fungal cell growth in chlorhexidine (CHX) group. (**C**–**E**) Different concentrations of CAF including 60, 30, and 15 mg/mL, respectively, showing the dose-dependent effects of CAF and the gradual growth inhibition of fungal cells. (**F**) CFU count of different SDA plates. Data are expressed as mean ± SD (*n* = 3) and statistically analyzed using Kruskal–Wallis test and Dunn’s post hoc test. The significance was considered when * *p* ≤ 0.05 as compared with control.

In Figure 2, agar plates of the CFU test to enumerate the fungal colonies for further confirmation showed overgrowth of *C. albicans* in the control PBS group with no treatments (Figure 2A). This *Candida* growth was reduced, as shown in SDA plates from the CHX group (Figure 2B). Caffeine groups in 60, 30, and 15 mg/mL concentrations demonstrated marked and dose-dependent decreased *C. albicans* colony count on the SDA plates (Figure 2C–E). The CFU counts in CHX and caffeine (60 mg/mL) groups were statistically significantly (*p* ≤ 0.05) low as compared to the control group (Figure 2F). Other concentrations of caffeine (30 and 15 mg/mL) showed a non-statistically significant lower CFU as compared to the control group (Figure 2F). Hence, caffeine at the 60 mg/mL concentration was considered as the MFC value as this concentration demonstrated less than 2 CFUs of *C. albicans.*

**Figure 3:**
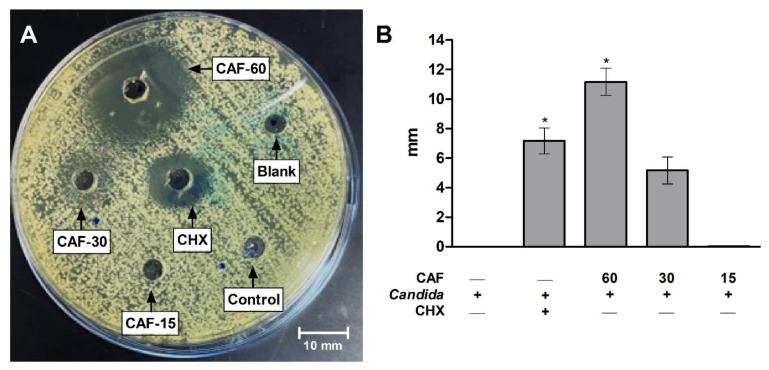
Agar-well diffusion method for caffeine (CAF) effects against *C. albicans* growth. (**A**) Different concentrations of CAF including 15 mg/mL, 30 mg/mL, and 60 mg/mL were tested. The higher two concentrations of CAF (30 and 60 mg/mL) showed the most prominent inhibition of the fungal growth as compared with control containing PBS alone, blank containing distilled water, and standard therapy containing chlorhexidine (CHX). (**B**) Measurements of zone of inhibition (mm). Data are expressed as mean ± SD (*n* = 3) and statistically analyzed using Kruskal–Wallis test and Dunn’s post hoc test. The significance was considered when * *p* ≤ 0.05 as compared with control.

The agar-well diffusion test on SDA plates demonstrated no growth inhibition zones in control (PBS), blank (distilled water), and caffeine (15 mg/mL) groups. On the other hand, CHX, caffeine (30 mg/mL) group, and caffeine (60 mg/mL) showed clear zones of *C. albicans* growth inhibition (Figure 3A). The inhibition zones on SDA plates presented as mm, and were significantly (*p* ≤ 0.05) higher in CHX and caffeine (60 mg/mL) groups as compared to the control group (Figure 3B). The 30 mg/mL caffeine concentration showed a non-statistically significant increase in the growth inhibition zones as compared to the control group (Figure 3B).

**Figure 4:**
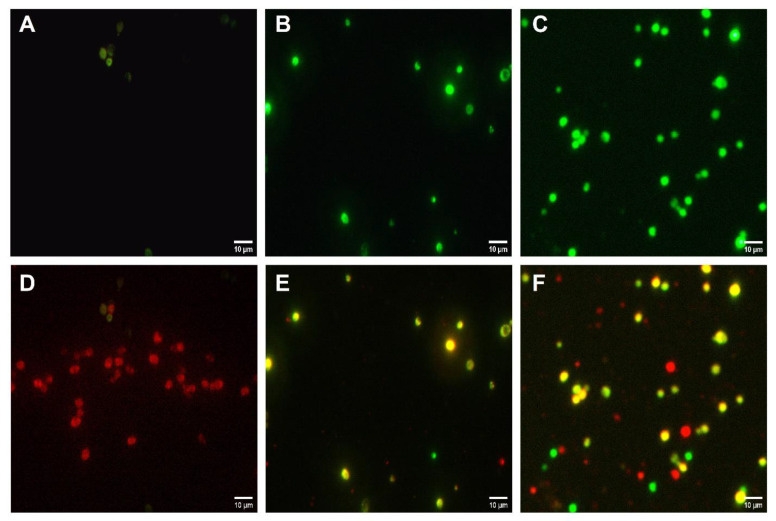
Live/Dead yeast viability method for caffeine (CAF) effects against *C. albicans* growth. The highest concentration of CAF (60 mg/mL) was tested. (**A**–**C**) Image showing dead fungal cells stained in green without treatments (**A**), with CHX treatment (**C**), and with CAF (60 mg/mL) treatment. (**D**–**F**) Image showing merged dead/live fungal cells stained in yellow-green or red-orange, respectively, after no treatments (**D**), after treatment with CHX (**E**), and after treatment with CAF (60 mg/mL) (**F**). Full plasma membrane integrity and metabolic function of yeast are needed to convert the yellow-green fluorescent into red-orange color.

The Live/Dead yeast viability test revealed the prominent effects of caffeine against *C. albicans* growth. The fluorescent microscope images showed a low number of fungal cells stained in light green with FUN-1 (Figure 4A), whereas a high number of cells stained red with Calcofluor White M2R were present (Figure 4D) in the control group without treatments. CHX group demonstrated higher dead fungal cells stained in green (Figure 4B) with markedly lower live red-stained cells (Figure 4E) when compared to the control group. The higher dose of caffeine (60 mg/mL) showed increased number of dead green-stained fungal cells (Figure 4C) and reduced count of the red-stained live cells (Figure 4F).

**Figure 5:**
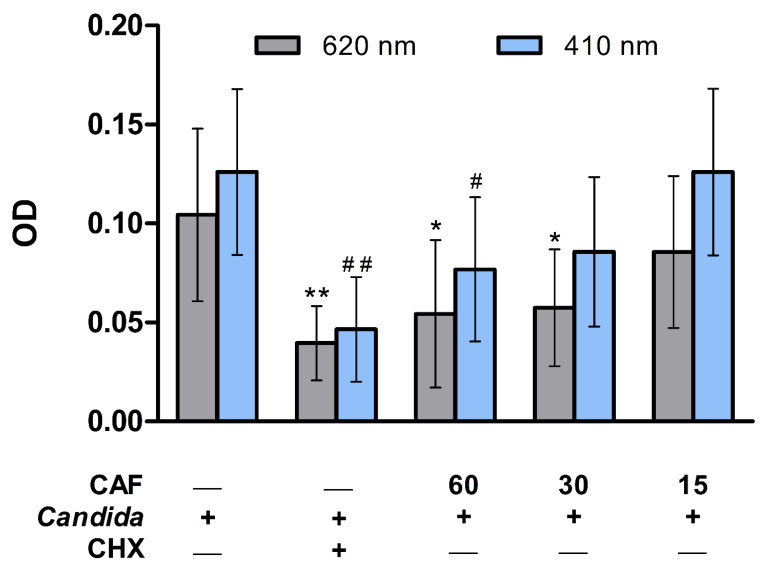
Microdilution test for caffeine (CAF) effects against the growth of *C. albicans* adherent to the PMMA discs showing the optical density (OD) of different groups measured at 620 nm and 410 nm. Data are expressed as mean ± SD (*n* = 10) and statistically analyzed using one-way ANOVA followed by Tukey’s post hoc test. The significance was considered when * *p* ≤ 0.05 and ** *p* ≤ 0.01 as compared with control at 620 nm or ^#^ *p* ≤ 0.05 and ^##^ *p* ≤ 0.01 as compared with control at 410 nm.

Caffeine’s effect was assessed against the adherent *C. albicans* to the PMMA discs using the microdilution test (Figure 5). The optical density (OD) measured at 620 nm or 410 nm revealed a significant (*p* ≤ 0.01) reduction in adherent *C. albicans* count in the CHX group as compared to the control group (Figure 5). Similarly, the higher dose of caffeine (60 mg/mL) showed a significant (*p* ≤ 0.05) decrease in adherent *C. albicans* count as compared to the control group at both weave lengths, whereas the 30 mg/mL dose significantly (*p* ≤ 0.05) reduced the attached fungal count compared to the control group when measured at 620 nm (Figure 5).

**Figure 6:**
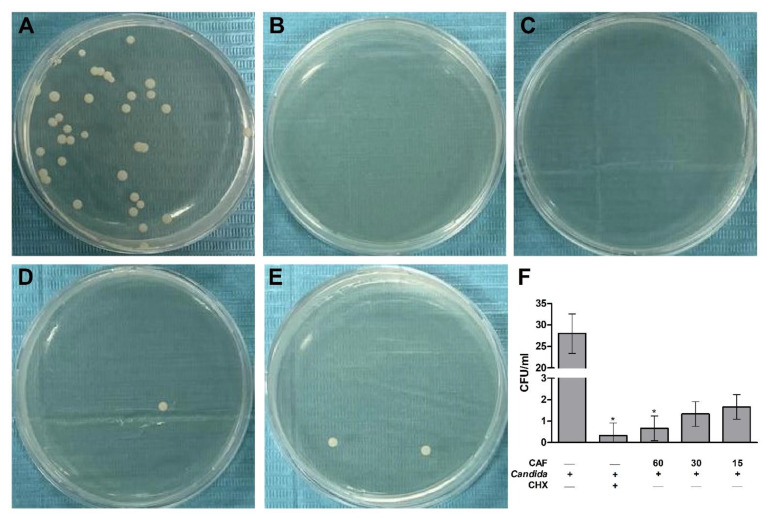
Colony-forming unit (CFU) method for caffeine (CAF) effects against the growth of *C. albicans* adherent to the PMMA discs. (**A**) Fungal cell growth in control group (PBS) containing no treatments. (**B**) Complete inhibition of the fungal cell growth in chlorhexidine (CHX) group. (**C**–**E**) Different descending concentrations of CAF including 60, 30, and 15 mg/mL, respectively, showing the dose-dependent effects of CAF and the gradual growth inhibition of fungal cells. (**F**) CFU count of different SDA plates. Data are expressed as mean ± SD (*n* = 3) and statistically analyzed using Kruskal–Wallis test and Dunn’s post hoc test. The significance was considered when * *p* ≤ 0.05 as compared with control.

SDA plates of the CFU test are presented in Figure 6. The control group with no treatments showed the highest adherent *C. albicans* colony count (Figure 6A), whereas other plates from CHX (Figure 6B) and caffeine groups (Figure 6C–E) demonstrated a markedly lower colony count. The adherent *C. albicans* colony count on the PMMA discs, presented as CFU/mL, was significantly (*p* ≤ 0.05) reduced by CHX and caffeine (60 mg/mL) treatments as compared to the control group (Figure 6F). Other concentrations of caffeine (30 and 15 mg/mL) showed a non-statistically significant reduction of the adherent *C. albicans* count as compared to the control group (Figure 6F).

**Figure 7:**
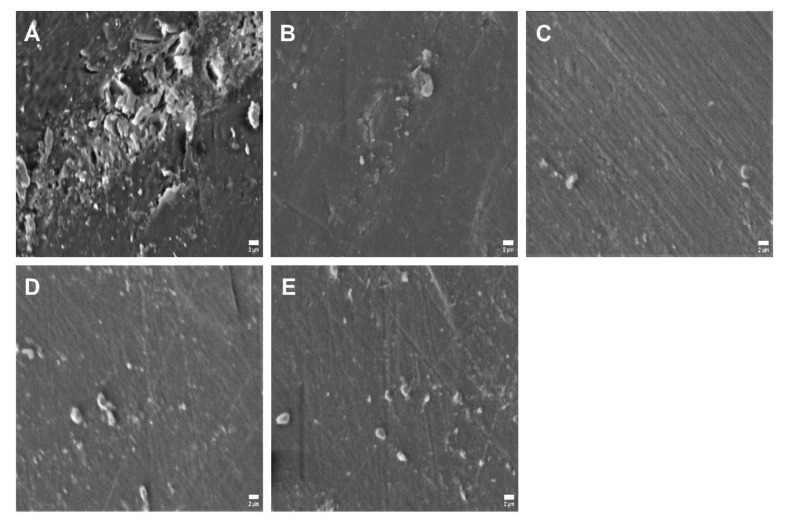
Scanning electron microscope (SEM) images showing the effects of caffeine (CAF) against the growth of *C. albicans* adherent to the PMMA discs at X5000. (**A**) Adherent fungal cells in control group (PBS) containing no treatments. (**B**) Limited number of the adherent fungal cells in chlorhexidine (CHX) group. (**C**–**E**) Different descending concentrations of CAF including 60, 30, and 15 mg/mL, respectively, showing the dose-dependent effects of CAF and the gradual reduction of the adherent fungal cells on the PMMA discs.

Results of the SEM revealed adherent *C. albicans* cells to the surface of PMMA discs in the control group (Figure 7A), whereas this number was markedly reduced in the standard therapy group by CHX (Figure 7B). Images of the SEM also showed a lower count for the *C. albicans* cells attached to the surface of PMMA discs in caffeine groups. The effect of caffeine was dose-dependent, at which the 60 mg/mL dose demonstrated the most prominent reduction in adherent fungal cell count (Figure 7C), which was equivalent to the CHX group compared to the control group. Other concentrations of caffeine (30 and 15 mg/mL) also reduced the adherent *C. albicans* count as compared to the control group (Figure 7D,E).

## 4. Discussion

*C. albicans* can be found in abundance on the surface of acrylic denture resin. Among the >700 microbial species that colonize the oral cavity, this fungus is considered the most common contributing factor in the pathogenesis of denture stomatitis [27]. Studies have reported that denture stomatitis is prevalent in almost 70% of denture wearers, which requires strict adherence to oral health instruction [28]. Accordingly, it could be anticipated that the daily consumed beverages might influence denture stomatitis and its caustic factors. Caffeine is a major component of coffee. It is also an active constituent in cocoa beverages, tea leaves, soft drinks, and chocolate-related products [29]. An average daily intake of 2 coffee cups is almost equivalent to 180 mg/d of caffeine [30]. The present study reinforced, using multiple techniques, that caffeine has a prominent direct antifungal property against *C. albicans.* Additionally, our findings demonstrated for the first time the influence of caffeine alone on *C. albicans* adherent to the PMMA acrylic denture resin discs, which was not documented in previous studies. PMMA is a globally utilized material in modern dentistry and is commonly used to fabricate dental prosthetics (such as dentures), artificial teeth, and orthodontic appliances. Therefore, PMMA discs represent the most suitable in vitro model for denture stomatitis [31]. Caffeine had antifungal activities and decreased *C. albicans* adhesion to PMMA denture base resin. Therefore, the null hypothesis was rejected.

In the present study, caffeine antifungal effects, particularly at the 60 mg/mL concentration, were comparable with the standard CHX therapy, indicating its prominent effects. Caffeine inhibited the growth of *C. albicans* after 24 and 48 h of incubation. The antimicrobial effects of caffeine are well-documented. On the fungal level, a wound dressing that delivers caffeine alone or in combination with ascorbic acid showed a clear antifungal activity against *C. albicans* using the disc diffusion method [32]. In one study, caffeine enhanced the antifungal activity of chloroquine through induction of the cell wall perturbation [33]. Caffeine and its salts caused alterations in the structure of *C. albicans* in the Mittag study [34]. Sabie and Gadd reported that caffeine and other phosphodiesterase inhibitors might induce yeast–mycelium transition of *C. albicans* [35]. Different concentrations of caffeine triggered gene segregations of *C. albicans* and suppressed its cell replication [36]. The reported direct antifungal effects of caffeine in the current study were confirmed by CFU assay and the agar well-diffusion method, where caffeine, particularly the 60 mg/mL concentration, suppressed the number of *C. albicans* colonies and generated a fungal growth inhibition zone wider than CHX. Therefore, the highest concentration of caffeine was chosen to innovatively verify its antifungal effects using the florescent yeast Live/Dead viability assay. In this test, the reduced count of the viable *Candida* indicated that caffeine could disturb the integrity of the fungal plasma membrane as well as induce alterations in the metabolic function of the yeast.

Multiple mechanistic pathways have been suggested to explain the antimicrobial effects of caffeine. For instance, caffeine might inhibit microbial DNA synthesis by suppressing thymidine kinase and preventing the inclusion of thymidine or adenine bases, which might additionally potentiate the effects of other antimicrobial agents [37,38]. Furthermore, the antimicrobial effects of caffeine might be indirectly augmented by its combination with other antimicrobial agents that target microbial cell-wall synthesis, which facilitates the influx of caffeine into microorganisms [39]. The antifungal effects of caffeine might be attributed to the manipulation of the N-terminal domain and specific protein phosphatase Z1 (CaPpz1) of *C. albicans* [40]. Thus, CaPpz1 gene deletion improved *C. albicans* sensitivity to caffeine, which supports this mode of action [41]. Studies have also suggested the role of CaTip41, CaSCH9, CaDOA1, and CDR1-yEGFP3 genes in regulating caffeine antifungal properties [42,43,44,45]. Stichternoth et al. reported that Tor1 and Sch9 kinases are essential for caffeine antifungal effects through suppression of hyphal morphogenesis [46]. Sanglard explored the calcineurin pathway as an essential tool for tolerance to *C. albicans* metabolic inhibitors such as caffeine [47].

One of the novelty aspects of the present work is that we tested the influence of caffeine alone against the adherent *C. albicans* on the surface of denture-based PMMA acrylic resin. One recent study conducted by Alfaifi et al. found that caffeine can suppress the metabolic activity and biofilm formation of *C. albicans* in the presence of nicotine on PMMA acrylic denture resin [20]. Nicotine was used to replicate the smoking condition as a causative factor for denture stomatitis. In the Alfaifi et al. study, the effect of caffeine alone against PMMA adherent *C. albicans* was not evaluated. Moreover, the range of caffeine concentrations in the Alfaifi et al. study was similar to our assessed caffeine concentrations. In the present study, caffeine markedly and dose-dependently reduced adherent *Candida* count as indicated by reduced OD at both 620 nm and 410 nm wavelengths. Here, we anticipated that the adherent *Candida* cell count would be low. Given the fact that the OD of *Candida* cell suspension could be recognized at different wavelengths [48,49], we inspected the OD of the adherent *Candida* at 620 nm and 410 nm wavelengths. Moreover, CFU assay results of the adherent *Candida* revealed the marked effects of caffeine against *Candida* biofilm formation at all tested concentrations. Treatment of the PMMA discs with different concentrations of caffeine lowered the number of surviving adherent *C. albicans*, which lowered OD values and CFU count of caffeine groups, particularly the higher (60 mg/mL) dose. SEM examination confirmed these findings. The SEM images were comparable to other studies, where *C. albicans* showed similar adhesion and biofilm formation without treatment on the PMMA discs. Indeed, the pattern of the reduced *Candida* adhesion by caffeine on SEM images resembled previous studies in its structure and arrangements [50,51].

The present work considered only the artificial saliva during the adherent *Candida* biofilm assay. The impact of salivary composition as well as pH was not studied. Additionally, the surface properties of the PMMA discs were not evaluated. Other surface properties or modified resin structures might influence the caffeine effects on the adherent *Candida* biofilm. The effect of pure caffeine was assessed in this study, which does not exactly mimic the condition case of daily beverages. Other excipients in the daily beverages should be also considered. These limitations might be considered in future research. Further studies are crucial to investigate the antifungal and antibiofilm effect of caffeine on different types of dentures used by edentulous patients and could be a promising solution to decrease the number of oral candidiasis cases. Moreover, in terms of denture base properties, the effects of caffeine with different concentrations on the strength of denture base resins are required.

## 5. Conclusions

The conscious use of a disinfectant is a fundamental element to prevent oral candidiasis infection. Taken together, the findings of the present in vitro study reinforced the antifungal properties of different caffeine concentrations against *C. albicans.* The 60 mg/mL concentration showed the most prominent effects that were comparable with standard CHX therapy. Caffeine also could reduce the antibiofilm activity of *C. albicans* and decrease the adhesion of the fungus on the PMMA acrylic denture base, which supports its potential use as an alternative disinfectant solution for fungal biofilms on denture surfaces.

## Data Availability

All data supporting the findings of this study are available within the manuscript.
